# A novel *TRPC6* mutation in a family with podocytopathy and clinical variability

**DOI:** 10.1186/1471-2369-14-104

**Published:** 2013-05-10

**Authors:** Amy K Mottl, Mei Lu, Catherine A Fine, Karen E Weck

**Affiliations:** 1 University of North Carolina Kidney Center, UNC School of Medicine, Chapel Hill, NC, USA; 2 Department of Pathology & Laboratory Medicine, University of North Carolina School of Medicine, Chapel Hill, NC, USA; 3 Department of Genetics, University of North Carolina School of Medicine, Chapel Hill, NC, USA

**Keywords:** Proteinuria, Minimal change disease, Focal segmental glomerulosclerosis, Genetic testing, TRPC6, Genotype-phenotype correlation

## Abstract

**Background:**

Mutation in several podocyte-specific genes have been noted to result in phenotypic heterogeneity. Herein, we report a novel, autosomal dominant *TRPC6* mutation in a family with disease ranging from asymptomatic minimal change disease to end-stage kidney disease.

**Case presentation:**

A 35 year old woman developed asymptomatic, nephrotic range proteinuria during pregnancy that did not resolve after delivery. Her mother had end-stage kidney disease of unknown etiology and her brother had asymptomatic proteinuria. Kidney biopsy revealed minimal change disease in both the proband and her brother. Genetic testing was performed in the proband and mother, revealing a novel frameshift mutation in *TRPC6*, D873fsX878. The proband continues to have subnephrotic range proteinuria and normal creatinine but her brother has since developed progressive chronic kidney disease.

**Conclusions:**

The current case report underscores the heterogeneity of disease in podocytopathies and related genes. Genetic testing of podocyte genes is useful in order to understand the pathophysiologic processes underlying these overlapping diseases.

## Background

Diseases of the podocyte most commonly manifest as minimal change disease (MCD) and focal segmental glomerulosclerosis (FSGS) in children and adults, respectively. A substantial proportion of both diseases are due to genetic aberrations of genes that are important to podocyte structure or function. The majority of the currently recognized genetic forms of MCD and FSGS follow simple Mendelian inheritance patterns [1]. Mutation in the podocyte-specific gene *TRPC6* was first identified as causing autosomal dominant (AD) FSGS in 2005 [2,3]. A total of 16 mutations in *TRPC6* have since been cited in both familial and sporadic FSGS as well as in adult and childhood onset disease [2-11]. To date, only a single family has been identified as having kidney histopathology other than FSGS [8]. Herein, we add to this body of literature by reporting a novel *TRPC6* mutation in a family with phenotypic heterogeneity ranging from asymptomatic minimal change disease to end-stage kidney disease (ESKD).

## Case presentation

A 34 year-old Korean woman was in her first trimester of pregnancy when she developed proteinuria. She was not edematous and did not have hypertension. Her proteinuria became increasingly severe and by her third trimester of pregnancy her urine protein to creatinine ratio (UPC) was greater than 10 gm/gm. She was induced at 37 weeks gestation and delivered a healthy boy who had no complications. Her proteinuria did not resolve following delivery and percutaneous kidney biopsy was performed. This demonstrated 1 out of 20 globally sclerotic glomeruli with no other abnormalities on light microscopy. Immunofluorescent staining was negative. Electron microscopy revealed moderate podocyte effacement and segmental basement membrane thinning. She has persistently declined treatment, including use of angiotensin converting enzyme inhibitors and all forms of immunomodulatory therapy. Despite this, her UPC has been stable for several years at 1.0 gm/gm and her creatinine has consistently measured less than 1.0 gm/dl.

**Figure 1:**
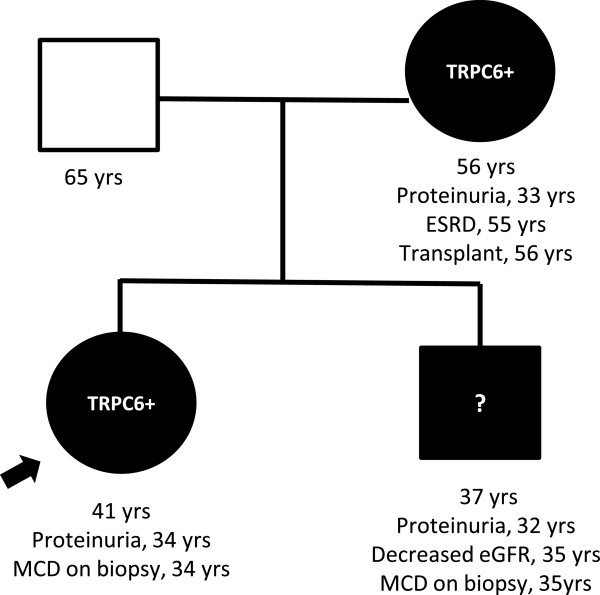
**Genealogical tree of a non-consanguineous, Korean family with minimal change disease and a novel *TRPC6 *deletion mutation in exon 12.**

There was suspicion of a genetic component to her disease as her mother had developed end-stage kidney disease in her mid-50s and her brother had proteinuria progressing to chronic kidney disease (Figure 1). The mother presented with shrunken kidneys and had not been able to undergo biopsy; she underwent kidney transplantation at 55 years and has had no proteinuria since that time. The brother’s clinical history has not been confirmed due to lack of consent. Per his sister’s report, proteinuria was initially mild and he opted against kidney biopsy until his proteinuria worsened and he subsequently developed chronic renal insufficiency. His biopsy reportedly demonstrated minimal change disease and he was treated with oral steroids without any improvement.

**Figure 2:**
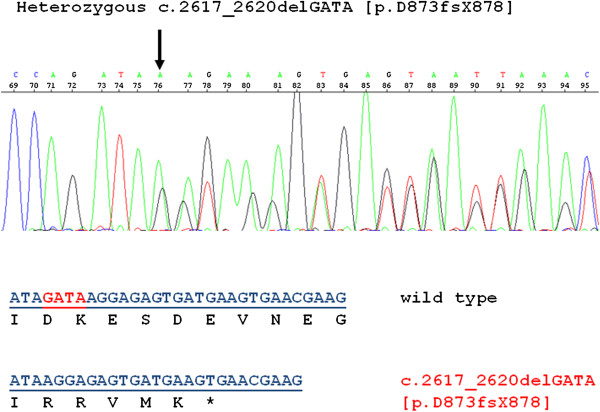
**Sequence analysis of the novel *TRPC6 *mutation found in 3 family members.** Shown is a partial electropherogram of Sanger DNA sequencing analysis of *TRPC6* exon12 from the proband. The arrow shows the position of the heterozygous four base pair deletion resulting in a downstream frameshift. Shown above are the nucleotide and predicted amino acid sequences of the wild-type *TRPC6* sequence (top line) and the heterozygous four base pair deletion (bottom line). The deleted nucleotides (GATA) are depicted in red font. The four base pair deletion results in a frame shift and premature protein truncation, five amino acids downstream [p.D873fsX878].

Genetic testing of *TRPC6* was performed due to the apparent autosomal dominant inheritance pattern. She was found to be heterozygous for a novel *TRPC6* frameshift mutation resulting from a 4 base pair GATA deletion in exon 12 [c. 2617–2620 del GATA, p. D873fsX878] (Figure 2). The reported mutation is novel, as it has not been seen in over 90 patients with nephrotic syndrome sequenced in our laboratory, is not reported in any dbSNP or exome sequencing databases of normal controls, and has not been previously reported in *TRPC6*. The resultant frameshift leads to a premature stop codon, and is predicted to result in a truncated TRPC6 protein lacking the C-terminal coiled coil domain. The proband’s mother also tested positive for the same mutation but her brother did not wish to be tested. DNA sequencing for mutations in *ACTN4, INF2,* and *NPHS2* associated with adolescent or adult onset nephrotic syndrome revealed no other mutations in this family (data not shown). Due to segmental basement membrane thinning on the proband’s kidney biopsy and the possibility of X-linked transmission in this family, the collagen 4 alpha 5 (*COL4A5*) gene was also sequenced in the proband’s mother and was negative for mutations.

## Conclusions

TRPC6 is a transient receptor potential (TRP) channel that plays a role in intracellular calcium signaling and is expressed in a signaling complex with nephrin and podocin in the podocyte slit diaphragm [1]. Although the reported *TRPC6* frameshift mutation is novel, it encodes a truncated TRPC6 protein lacking the C-terminal coiled coil domain, a highly conserved domain thought to play an essential role in TRPC6 calcium channel function. This truncating mutation is likely to have a dominant gain-of-function effect similar to other known pathogenic *TRPC6* mutations (Table 1) [3,6]. Other *TRPC6* mutations mapped to the coiled-coil domain at the C-terminus, such as R895C and E897K, have been demonstrated to result in increased calcium ion influx [3,6]. The C-terminal K874X nonsense mutation and N-terminal mutations within the ankyrin repeats have demonstrated delayed calcium channel inactivation, resulting in increased channel opening time [5,12]. The D873fsX878 mutation results in protein truncation only four amino acids downstream of the truncating K874X mutation and may have a similar effect on delaying calcium channel inactivation. The tracking of this mutation with nephrotic syndrome in this family supports an autosomal dominant gain of function effect. The mechanism whereby increased calcium current leads to the pathogenic manifestations of MCD or FSGS is unknown. TRPC6 functions as a mechanoreceptor of membrane stretch [1]. Other pathogenic mechanisms of *TRPC6* mutation that have been hypothesized include altered channel regulation, altered interaction with other slit diaphragm proteins, and altered protein turnover [3]. It has been speculated that mutations in *TRPC6* may result in apoptosis, podocyte detachment or an alteration in the ultrafiltration coefficient [12,13].

**Table 1:** Mutations in TRPC6 protein currently identified to cause proteinuric kidney disease

**TRPC6 mutation**	**Effect on ion channel function**	**Level of evidence**	**Ethnicity**	**Phenotype**	**Age at presentation (years)**	**Reference**
89fsX8	Not evaluated		Caucasian	FSGS	7	[7]
G109S	Probably damaging	In silico scoring matrix	Caucasian	FSGS	25	[6]
N125S	Probably damaging	In silico scoring matrix	Caucasian	sporadic FSGS	41	[6]
	Increased intracellular calcium	In Vitro experiments	Caucasian	MCD and IgAN with MPGN-like pattern	4-14	[8]
M132T	Increased current amplitude and delayed channel inactivation	In Vitro experiments	Caucasian	AD FSGS	9-30	[5]
	Not evaluated		Caucasian	FSGS	8	[7]
P112Q	Increased current amplitude	In Vitro experiments	Caucasian	AD FSGS	30-40	[2]
N143S	None identified	In Vitro experiments	African American	AD FSGS	30-40	[3]
	None identified	In Vitro experiments	Caucasian	AD FSGS	27-39	[5]
H218L	Increased intracellular calcium	In Vitro experiments	Caucasian	sporadic FSGS	8	[8]
S270T	None identified	In Vitro experiments	Latino	AD FSGS	20-50	[3]
R360H	Not evaluated		Not stated	FSGS	34	[11]
L395A	Not evaluated		Caucasian	sporadic FSGS	2	[10]
G757D	Not evaluated		Caucasian	FSGS	1	[7]
L780P	Possibly damaging	In silico scoring matrix	Caucasian	sporadic FSGS	7	[6]
D873fsX878	Not evaluated			MCD	34-50	Present study
K874X	None identified	In Vitro experiments	Caucasian	AD FSGS	30-60	[3]
Q889K	Increased current amplitude	In Vitro experiments	Chinese	AD FSGS	>12	[4]
R895C	Increased current amplitude	In Vitro experiments	Latino	AD FSGS	20-50	[3]
	Not evaluated		Caucasian	AD collapsing FSGS	21-38	[9]
R895L	Increased intracellular calcium	In Vitro experiments	Caucasian	sporadic collapsing FSGS	1	[8]
E897K	Increased current amplitude	In Vitro experiments	Caucasian	AD FSGS	25-35	[3]

AD-Autosomal Dominant; FSGS-Focal Segmental Glomerulosclerosis; MCD-Minimal Change Disease; IgAN-IgA Nephropathy; MPGN-Membranoproliferative Glomerulosclerosis.

To date, the vast majority of identified mutations in *TRPC6* have been associated with FSGS, including collapsing glomerulopathy [2-8,10,11]. The current report underscores the phenotypic heterogeneity that can occur in podocyte-specific gene mutations. We have replicated the finding that *TRPC6* mutation can result in MCD as well as FSGS [8]. Gigante et al. (2011) previously reported a family with a distinct *TRPC6* mutation resulting in childhood onset nephrotic syndrome, with one sibling having MCD and another having IgAN with a membranoproliferative-like pattern. In some cases, the finding of MCD is due to unsampled FSGS and in other instances it may represent an earlier clinical stage of FSGS, which could be true in the present case. *TRPC6* mutation has traditionally been thought to cause familial, adult onset disease; however, a significant number of sporadic and childhood onset FSGS cases have been identified [5-8]. Other podocyte-specific genes have also been found to result in multiple pathologic and clinical disease phenotypes. Mutations in podocin (*NPHS2*) have been reported in FSGS, steroid resistant MCD and diffuse mesangial sclerosis [14]. Phenotypic heterogeneity also exists for nephrin (*NPHS1*) mutations which have been reported to result in congenital nephrotic syndrome, steroid-resistant MCD and FSGS [15]. Given the heterogeneity in disease that can result from mutations within the same gene, it has been suggested that these disease entities be referred to as ‘podocytopathies’ rather than by their histologic phenotype [16].

This family demonstrates the significant heterogeneity in clinical manifestations that can occur from the same genetic mutation. Although genotype-phenotype correlation of this novel *TRPC6* mutation is not known, all three affected adults presented with proteinuria at 32–34 years of age, with progressive chronic kidney disease subsequently developing in the proband’s mother and brother. However, the proband has thus far remained stable following pregnancy-induced proteinuria without any increase in creatinine. There are likely other genetic and/or environmental modifiers that play a significant role in disease penetrance and expressivity. Currently, there are no known genetic modifiers of *TRPC6*-associated disease. Modifiers for *TRPC6*-mediated podocytopathies can be speculated when considering that TRPC6 is partially regulated by podocin and is thought to be assembled in a complex with nephrin. The R229Q single nucleotide polymorphism in podocin (*NPHS2)* results in decreased nephrin binding and can result in both familial and seemingly sporadic FSGS when in a compound heterozygous state with another deleterious *NPHS2* mutation [17]. Heterozygous lesions in collagen IV have also been hypothesized to modify susceptibility to FSGS [18]. Although sequence analysis of both *NPHS2* and the X-linked collagen IV chain gene *COL4A5* was negative in this family, other genetic or environmental factors may play a role in modifying disease severity within a family and in determining whether *TRPC6* mutation presents as FSGS or MCD in different families.

This case report underscores the complexity in nomenclature, pathogenesis and genetics of podocytopathies. The clinical presentation, histological pattern, causative genes and specific mutations should all be taken into consideration when assessing a familial or even seemingly sporadic case of proteinuria. Documentation of atypical cases of familial proteinuric syndromes along with technological advances in genetic testing will facilitate a better understanding of diseases of the podocyte.

## Consent

Written, informed consent was obtained from the patient for publication of this case report.

## Abbreviations

MCD: Minimal Change Disease; FSGS: Focal Segmental Glomerulosclerosis; ESKD: End-Stage Kidney Disease.

## Competing interests

The authors declare that they have no competing interests.

## Authors’ contributions

AKM and CAF provided care to the proband. ML and KEW were responsible for genetic testing. AKM wrote the manuscript. All authors held discussions regarding the content of the manuscript and approved the final version of the manuscript.

## Pre-publication history

The pre-publication history for this paper can be accessed here:

http://www.biomedcentral.com/1471-2369/14/104/prepub
